# Mixed Cultures of *Saccharomyces kudravzevii* and *S. cerevisiae* Modify the Fermentation Process and Improve the Aroma Profile of Semi-Sweet White Wines

**DOI:** 10.3390/molecules27217478

**Published:** 2022-11-02

**Authors:** Urszula Błaszczyk, Paweł Satora, Łukasz Noga

**Affiliations:** Department of Fermentation Technology and Microbiology, Faculty of Food Technology, University of Agriculture in Krakow, Aleja Mickiewicza 21, 31-120 Krakow, Poland

**Keywords:** alcoholic fermentation, mixed cultures, *Saccharomyces kudriavzevii*, *Saccharomyces cerevisiae*, volatile compounds

## Abstract

The purpose of the study was to evaluate the impact of the *Saccharomyces cerevisiae* and *S. kudriavzevii* mixed culture on the fermentation, chemical and aromatic composition of semi-sweet white wines. The variables tested in the experiment were the initial ratio of yeast in mixed cultures and the time of inoculation of the *S. kudriavzevii* co-culture. The addition of *S. kudriavzevii* to the inoculum did not significantly change the chemical composition of the wines obtained. No reduction in ethanol yield was found in mixed culture fermented wines; however, in some variants of the experiment, the ethanol content was higher. The mixed cultures of *S. cerevisiae* and *S. kudriavzevii* increased the level of volatile compounds in white grape wines. Wines fermented with the co-culture of *S. kudriavzevii* were characterized by a more diversified ester profile. The mixed cultures of *S. cerevisiae* and *S. kudriavzevii* raised the levels of terpenes in white wines. The most promising results were obtained for mixed culture variants, in which *S. kudriavzevii* was sequentially inoculated on the sixth day of fermentation.

## 1. Introduction

Due to its high fermentation capacity, *Saccharomyces cerevisiae* is the yeast species that is traditionally used in most alcoholic fermentation processes, including wine fermentation. However, to meet specific challenges related to climatic change and consumer expectations, such as the search for wines with new unique sensory characteristics, altered alcohol and glycerol concentrations, selection and the use of alternative yeast species with desirable properties are required in vinification [1]. *Saccharomyces kudriavzevii* is one of the non-conventional species of *Saccharomyces* that could be considered as a new starter culture for wine production.

The *S. kudriavzevii* strains have been isolated from natural habitats such as soil and decaying leaves in Japan [2] and from oak barks in Portugal and Spain [3,4]. Interestingly, the distribution of the *S. kudriavzevii* species appears to be restricted to only two continents (Asia and Europe) as it has not been isolated from other regions such as North and South America [5]. *S. kudriavzevii* is not as widespread in fermentation processes as its hybrids with *S. cerevisiae*. Genetic analysis of *Saccharomyces* strains isolated from wine, beer and cider have revealed the presence of natural hybrids of *S. cerevisiae* × *S. kudriavzevii*, including a triple hybrid *S. cerevisiae* × *S. kudriavzevii* × *S. uvarum* [1,6,7]. Hybrids of *S. cerevisiae* × *S. kudriavzevii* have also been found in clinical samples and in dietary supplements [1]. As it turns out, hybrids with a higher proportion of the *S. cerevisiae* subgenome are better suited to fermentation stresses, while hybrids with a higher proportion of the *S. kudriavzevii* subgenome are more efficient at low-temperature fermentation [1,8]. Natural hybrids between *S. kudriavzveii* and *S. cerevisiae* have been shown to produce greater amounts of glycerol and higher alcohols than reference strains of their parent species [9].

As was shown from previous work [8,10,11], an interesting characteristic of *S. kudriavzevii* strains is a better adaptation to life at low temperatures than *S. cerevisiae* strains. *S. kudriavzevii* exhibits a good fermentation capability under these conditions, so it can be a good alternative for cold fermentations [12]. In addition, some strains of *S. kudriavzevii* have been shown to produce large amounts of glycerol [10,13] and higher alcohols, such as 2-phenylethanol [14,15]. The *S. kudriavzevii* strains can be used in the production of wine with lower ethanol and higher glycerol content [13].

The aim of this study was to determine the effect of mixed yeast cultures of two strains of the species *S. cerevisiae* and *S. kudriavzevii*, inoculated simultaneously and sequentially, on the fermentation process and the formation of selected volatile compounds during the fermentation of white wines.

## 2. Results and Discussion

### 2.1. Effect of Mono- and Mixed Cultures of S. cerevisiae and S. kudriavzevii on the Fermentation and Enological Parameters of White Wines

**Figure 1:**
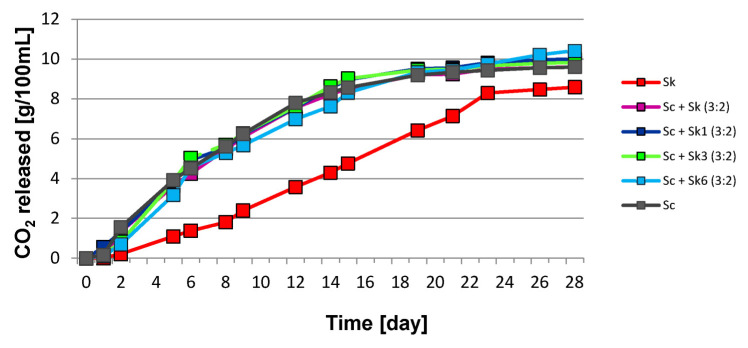
The kinetics of grape must fermentation with mixed cultures of *Saccharomyces cerevisiae* (Sc) and *S. kudriavzevii* (Sk) with an initial yeast ratio 3:2. Sk_1_, Sk_3_, Sk_6_: sequential inoculation of Sc followed Sk after one day, three days, six days.

**Figure 2:**
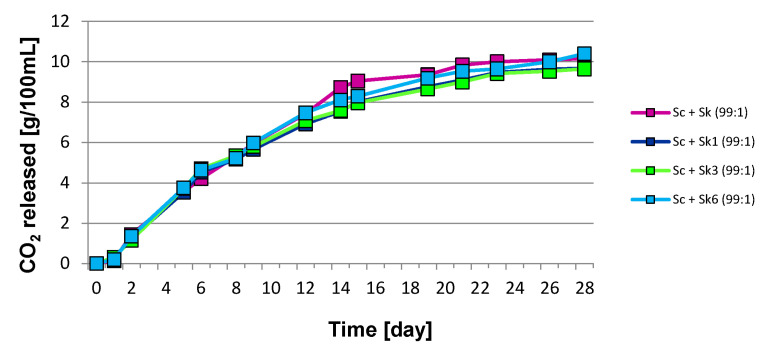
The kinetics of grape must fermentation with mixed cultures of *S. cerevisiae* (Sc) and *S. kudriavzevii* (Sk) with an initial yeast ratio 99:1.

The effect of mono- and mixed-cultures of *S. cerevisiae* and *S. kudriavzevii* simultaneously or sequentially inoculated on the dynamics of the fermentation process have been determined. The differences in the kinetics of fermentations depending on the time of inoculation of *S. kudriavzevii* and the initial proportion of yeast strains tested in the inoculums are shown in Figure 1 and Figure 2. Alcoholic fermentation was carried out for 28 days at a temperature of 20 °C. The largest weight losses associated with the release of carbon dioxide were observed during the first days of fermentation. After 28 days of the experiment, the final amount of liberated carbon dioxide was similar for all wines fermented with the *S. cerevisiae* monoculture and co-cultures with *S. kudriavzevii*. The greatest final weight losses were found in samples fermented with mixed cultures, in which *S. kudriavzevii* was sequentially inoculated on the sixth day after the inoculation of *S. cerevisiae*. The weakest fermentation rate was observed in samples inoculated with the *S. kudriavzevii* monoculture (Figure 1 and Figure 2).

Due to the differences in the course of fermentations, depending on the use of mono- and mixed cultures, inoculated simultaneously or sequentially, the wine samples were characterized by significantly differentiated ethanol yield. The ethanol content in the wines analyzed ranged from 8.7 to 11.2% *v*/*v* (Table 1). The highest concentrations of ethyl alcohol were observed in wines sequentially inoculated with *S. kudriavzevii* on the sixth day after inoculation with *S. cerevisiae*.

A slightly lower ethanol yield was observed in samples fermented with a mixed culture, in which *S. kudriavzevii* was inoculated three days after inoculation with *S. cerevisiae* (Table 1). The lowest ethanol content produced was found in the samples fermented with the pure culture of *S. kudriavzevii* (8.7%). This observation is in accordance with results reported by other authors. For example, studies on the modeling of wine fermentation by two species of yeast *Saccharomyces* (*S. cerevisiae* T73 and *S. kudriavzevii* CR85) at different processing temperatures carried out by Henriques et al. [12] also showed that fermentations with *S. kudriavzevii* CR85 were typically slower and produced less amount of ethanol.

One of the reasons for using mixed cultures of *S. cerevisiae* and non-*Saccharomyces* yeast or *Saccharomyces* non-*cerevisiae* yeast strain in fermentation can be to obtain wines with lower ethanol content. The use of a mixed culture with the *S. kurdriavzevii* strain in fermentation was expected to reduce the ethanol content. In our experiment, mixed fermentations did not lower the ethanol content compared to fermentations with pure *S. cerevisiae* culture. Only in the variant of the experiment using simultaneous inoculation of *S. cerevisiae* and *S. kudriavzvevii* with an initial ratio of 3:2, a slight reduction in ethanol yield was observed. In the case of wines obtained by sequential inoculation with co-culture on the sixth day, a slightly higher ethanol content was observed. The results of our research indicate that *S. cerevisiae* yeast dominated wine fermentations carried out at a temperature of 20 °C in the analyzed samples. Research conducted by Arroyo-López et al. [16] demonstrated that for both tested yeast strains (*S. cerevisiae* T73 and *S. kudriavzevii* IFO 1802^T^), a reduction in their maximum specific growth rates was observed in mixed fermentations, clearly showing an antagonism between the two tested micro-organisms. The authors noted that both ethanol and killer factors had no significant effect on the competition between *S. cerevisiae* and *S. kudriavzevii*, while temperature played the most important role. At a temperature of 31 °C, *S. cerevisiae* was the best competitor, while at low temperatures (17 °C) *S. kudriavzevii* grew faster than *S. cerevisiae* in the early stages of fermentation, when the inoculum ratio was 1:1. However, the growth of *S. kudriavzevii* was interrupted earlier than that of *S. cerevisiae* at any temperature tested. The authors indicated that these results could explain why *S. kudriavzevii* has not been found in the wine fermentation environment. So far *S. kudriavzevii* has not been isolated from vineyards, wineries, or fermenting wine [14,16]. In another study by Alonso-del-Real et al. [17] increased competitiveness of *S. kudriavzevii* CR85 was observed only in the case of co-inoculations with a low proportion of *S. cerevisiae* (<10%). The effect was enhanced when aeration (20 VVH) was used during fermentation, which resulted in a reduced ethanol yield and increased glycerol production. The results of the studies by Alonso-del-Real et al. [18], in a comparative transcriptomic analysis during fermentation with a mixed culture of *S. cerevisiae* and *S. kudriavzevii*, led to the conclusion that *S. kudriavzevii* demonstrated a reaction to competition, but this reaction in *S. kudriavzevii* was delayed and weaker than in *S. cerevisiae*, which accelerated the uptake and utilization of nutrients to combat the co-inoculated yeast strain. It has also been noted that this process required cell-to-cell contact, which is an important condition for wine yeast to overcome its competitors.

The total extract content in the tested wines ranged from 29.6 to 69.8 g/L (Table 1). A decrease in the total extract content was proportional to the amount of ethanol produced during the fermentation process. A similar tendency was observed for total and reducing sugars. The grape musts that were sequentially inoculated with the *S. kudriavzevii* DSM 3774 strain on the first, third and sixth day of fermentation were characterized by lower amounts of residual sugars compared to the trials inoculated simultaneously and monocultures. The obtained wines were classified as semi-sweet (up to 45 g/L sugars). The lowest level of sugar utilization was demonstrated during fermentation with the pure culture of *S. kudriavzevii*, which fermented 84% of the reducing sugars initially present in the must. The highest concentration of unfermented sucrose, similar to that of reducing sugars, remained in wines fermented with the *S. kudriavzevii* monoculture. Wines obtained with pure *S. cerevisiae* Johannisberg Riesling ŁOCK 105 culture, as well as mixed cultures inoculated simultaneously, had a slightly lower sucrose content, while in beverages obtained by sequential fermentation, sucrose concentrations were significantly reduced. The sequential addition of *S. kudriavzevii* co-culture on the sixth day of fermentation resulted in the complete use of this disaccharide.

The glycerol concentration in the wines tested varied between 6.4 and 8.1 g/L (Table 1). Samples fermented with the pure culture of *S. cerevisiae* were characterized by the highest glycerol content. Similar results were observed for the samples fermented with mixed culture simultaneously inoculated with Sc and Sk, in which the inoculum ratio of Sc and Sk was 99:1. In other cases, the use of mixed yeast cultures resulted in a slight reduction in glycerol level compared to wine fermented with the monoculture of *S. cerevisiae*. Glycerol concentrations in wines generally range between 4 and 9 g/L, with average values approximately of 7 g/L [19]. Glycerol significantly contributes to wine quality by providing slight sweetness, fullness, and smoothness of the taste [19]. The results of earlier studies have shown that the non-wine yeast *S. kudriavzevii* IFO 1802^T^ produced higher glycerol levels and lower ethanol content than the wine strains *S. cerevisiae* and their hybrid W27, which was consistent with the increased activity of glycerol-3-phosphate dehydrogenase [20]. Increased glycerol production was also observed in the experiment of Henriques et al. [12], in which the *S. kudriavzevii* CR85 strain was used for fermentation. The results of our study showed that during fermentation at 20 °C, the glycerol production by the *S. kudriavzevii* DSM 3774 monoculture or co-cultures was not higher than that of the pure *S. cerevisiae* strain, which may be an individual characteristic of the *S. kudriavzevii* strain used in our experiment.

Wines obtained as a result of fermentation with a pure culture of *S. kudriavzevii* were characterized by higher titratable acidity (10.1 g/L) compared to the other samples. A similar trend was observed for volatile acidity. Wines fermented with *S. kudriavzevii* DSM 3774 monocultures contained higher amounts of acetic acid (0.32 g/L). The use of mixed cultures or *S. cerevisiae* monoculture resulted in a decrease in the level of volatiles and total acidity.

### 2.2. Aroma Compounds and Sensory Analyzes

The application of mixed starters containing *S. cerevisiae* and selected non-*Saccharomyces* or *Saccharomyces* non-*cerevisiae* yeast can allow more diverse wines with an enriched aroma to be obtained [21,22].

Yeast strains grown in mixed cultures can metabolically interact with each other and thus modify the fermentation products. Certain compounds produced by one yeast strain can be taken up and used by another yeast strain in the co-culture [23]. Therefore, the chemical and sensory profiles of co-inoculated wines can be modified. This explains why the taste of wine obtained by mixed culture fermentation cannot be reproduced simply by blending wines fermented by a single strain and that the modification of the flavor of the wine is due to complex interactions between yeast strains in mixed culture [23,24]. Yeast strains in co-culture can influence (positively or negatively) the aroma profile of the wine by adding secondary metabolites produced by each yeast strain present in mixed culture. Another mechanism is based on specific metabolic interactions, i.e., enzymatic activity caused by the production of specific proteins modifying some grape-derived compounds [25].

The concentrations of selected volatile compounds in wines obtained by the fermentation of grape must with the participation of monocultures and mixed yeast cultures are presented in Table 2 and Table 3. The volatile esters content in the wine samples ranged from 264.0 to 316.8 mg/L (Table 2). Esters produced during wine fermentation are believed to be important compounds of the bouquet of the wine. The synthesis of esters during fermentation depends on the characteristics of the yeast strain, the composition of the medium, and the fermentation conditions [26]. The samples of the tested wines had an ethyl acetate concentration not exceeding 100 mg/L (Table 2). The use of sequential inoculation of *S. cerevisiae* and *S. kudriavzevii* strains increased the amount of this ester, which can positively influence the quality of the beverages obtained. A similar trend was also observed during sequential mixed fermentation carried out by *S. cerevisiae* Y3401 followed by *Wickerhamomyces anomalus* Y3604 [27]. Ethyl acetate, the most common ester in wine, at low levels (50–80 mg/L) can contribute to the olfactory complexity of a wine and thus has a positive effect on quality, however, at a concentration of 150–200 mg/L it can adversely affect the taste of the wine [28]. There were no significant differences in the content of isoamyl acetate and ethyl caproate between the samples analyzed (Table 2). Wines obtained by fermentation of musts inoculated sequentially with *S. kudriavzevii* on the sixth day after inoculation with *S. cerevisiae* contained increased amounts of most of the esters analyzed, including ethyl propanoate (sweet, ethereal, fruity-grape and pineapple aromas), ethyl 2-methylbutanoate (fruity, fresh, berry, grape and pineapple notes), ethyl 2,4-hexadienoate (ethereal, fruity odors) or ethyl hexadecanoate (fruity, apricot, sour cherry, bilberry, grapefruit, melon, pineapple scents) (Table 3). The availability of precursors is a limiting factor in the synthesis of ethyl esters. Thus, the rate of ethyl esters formation depends on the concentration of substrates and the activity of enzymes responsible for their synthesis and hydrolysis. Saerens et al. [29] found that the supply of MFCA to the fermentation medium increased the production of ethyl esters. The opposite effect has been reported with the use of unsaturated fatty acids. The initial nitrogen content, temperature, and lipid content were found to be other factors influencing the production of ethyl esters such as ethyl hexanoate or ethyl octanoate [26].

Intensification of ester production was also observed in the study of Renault et al. [32]. Sequential and simultaneous inoculation of mixed cultures of *Torulaspora delbrueckii* and *S. cerevisiae* also increased the level of esters in wines compared to fermentation with pure cultures. Some of these esters, such as ethyl propanoate, ethyl isobutanoate, or ethyl dihydrocinnamate, were specifically produced by *T. delbrueckii*, and their concentration was clearly correlated with the maximum population of *T. delbrueckii* during fermentation. Sequential inoculation (*T. delbrueckii* inoculated 24 h before *S. cerevisiae* yeast in a ratio 5:1) favored intensification of ester production related to the activity of *T. delbrueckii*. On the other hand, there was also a marked increase in other esters such as isobutyl acetate and isoamyl acetate, although their concentration was not closely correlated with the development of any yeast species used in the experiment. The level of these esters increased as a result of the positive interactions between *T. delbrueckii* and *S. cerevisiae*. The increase in isoamyl acetate production was caused by *S. cerevisiae* in response to the presence of *T. delbrueckii*. A similar trend was also observed with regard to phenylethyl acetate, ethyl butanoate, and ethyl decanoate, which were also produced at the highest concentrations in a simultaneous mixed method [32].

The carbonyl component content of wines is generally less than 100 mg/L [33]. This level was not exceeded in the wine samples analyzed (Table 2). The highest concentration of these compounds was found in samples with the *S. kudriavzevii* monoculture, while the wines fermented with pure *S. cerevisiae* culture contained smaller amounts of these compounds. The analyses of the tested beverages also showed that the subsequent addition of co-culture led to the increased synthesis of carbonyl compounds.

Acetaldehyde is the main carbonyl compound in wine. Acetaldehyde accounts for 90% of the total aldehyde content of the wine, of which only free acetaldehyde is of any importance in relation to the aroma of the wine. At high levels (>200 mg/L) acetaldehyde can have a detrimental influence on the aroma of wine, while at lower concentrations it can contribute to the fruity and nutty character of wine [34]. The samples tested were characterized by a rather low concentration of this compound (Table 2). The use of mixed cultures increased the formation of acetaldehyde, the highest level was found in samples to which the *S. kudriavzevii* strain was inoculated on the sixth day of fermentation and the initial yeast ratio was 3:2 (23.1 mg/L).

In addition to ethanol, alcohols such as isobutanol, amyl alcohols, n-propanol and 2-phenyloethanol are commonly found in wines. These compounds are released into wine as a product of the secondary metabolism of yeast, either directly from sugars or from grape amino acids by the Ehrlich reaction [28]. At low concentrations (less than 300 mg/L), they can have a positive effect on bouquet and to the aromatic complexity of a wine. In higher concentrations, they may be responsible for the spirit flavor [28]. *S. cerevisiae* strains are capable of synthesizing higher amounts of fusels compared to yeast such as *Candida*, *Kloeckera* or *Brettanomyces* [35]. The fusel content in the analyzed wine samples did not exceed 180 mg/L (Table 2). The use of *S. kudriavzevii* strains during vinification may contribute to the creation of a new aroma composition of wines, by modifying the content of higher alcohols and esters. In our experiment the highest content of higher alcohols was observed for the variant in which sequential inoculation was used for fermentation, in the ratio of *S. cerevisiae* to *S. kudriavzevii* 3:2, and the *S. kudriavzevii* strain was introduced 6 days after *S. cerevisiae* inoculation. The results of the study conducted by Stribny et al. [15] showed that at 12 °C, *S. kudriavzevii* can produce higher levels of fusel alcohols including 2-phenylethanol, which is related to its amino acid metabolism being different from that of *S. cerevisiae*. These differences in aroma compound production are correlated with differences in gene regulation [16]. *S. kudriavzevii* has been reported to modify the regulation of genes involved in the formation of ethyl esters during fermentation at 28 °C. The *S. kudriavzevii* strain presented upregulation of EHT1 acyltransferase and downregulation of EEB1 acyltransferase [36]. In contrast to other higher alcohols, propanol is formed by condensation of pyruvic acid and acetyl CoA [37]. This compound was present in the wine samples analyzed at a similar level (48.5 to 68.1 mg/L). Slightly higher propanol concentrations were observed in the samples in which *S. kudriavzevii* was added to the grape musts on the sixth day after inoculation with *S. cerevisiae* and the inoculum ratio was 3:2. The isobutanol content in wines ranges from 35 to 180 mg/L [38]. In wines tested it was found in relatively small amounts (from 31.4 to 40.4 mg/L). The wines fermented with monocultures of *S. kudriavzevii* or *S. cerevisiae* were characterized by similar amounts of amyl alcohols (68.8 mg/L). There were slight differences in the amyl alcohols content between the analyzed samples.

**Figure 3:**
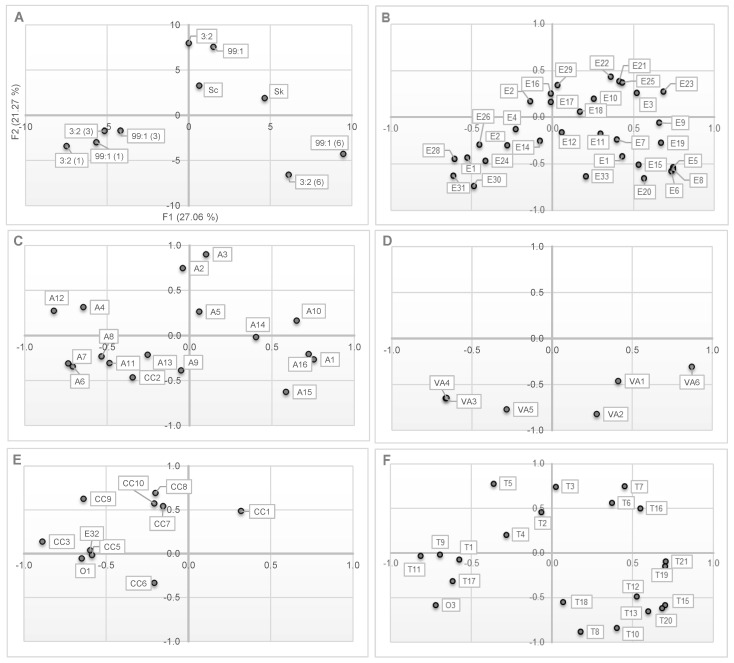
PCA plots based on SPME-GC-MS analysis of wine samples fermented with monocultures or mixed cultures of *Saccharomyces cerevisiae* (Sc) and *S. kudriavzevii* (Sk). (**A**) Observations; (**B**) Esters; (**C**) Alcohols; (**D**) Volatile Acids; (**E**) Carbonyl Compounds; (**F**) Terpenes.

Figure 3 presents the PCA results determined on the basis of SPME-GC-MS analysis in order to emphasize the differences in volatile compounds. The PCA results showed that the PC1 and PC2 biplots represented 48.32% of the total variance in the data set. Three distinct regions can be distinguished in the PCA profile (Figure 3A Observations). The first area (top right, Figure 3A) includes wine samples obtained by fermentation with monocultures or mixed cultures with simultaneous inoculation of both yeast strains tested with simultaneous inoculation of both yeast strains tested. The second area (bottom left, Figure 3A) is for the samples co-inoculated sequentially with the *S. kudriavzevii* DSM 3774 strain on the first or third day of fermentation. The third area (bottom right, Figure 3A) includes wine samples inoculated sequentially with *S. kudriavzevii* on the sixth day after the inoculation of *S. cerevisiae*. PCA analysis shows that the observed differences in the composition of volatile components of the obtained white wines were mainly influenced by the co-inoculation time with *S. kudravzevii*, while the ratio of *S. kudravzevii* in the inoculum did not play a significant role. The mixed cultures of *S. cerevisiae* and *S. kudriavzevii* used in this study increased the level of volatile compounds, including terpenes. In particular, the introduction of *S. kudriavzevii* on the sixth day of fermentation clearly contributed to the increase in variety and the level of terpenes in the wines obtained (Figure 3F Terpenes). The source of terpenoids in wines is grapes, in which these compounds exist free and as glycosylated conjugates [39]. The release of glycosylated compounds (e.g., monoterpenes) occurs by either chemical or enzymatic hydrolysis. Differences in the terpene profile of wines may depend on the activity of β-glucosidase as well as the rate of terpene bioconversion and the percentage of accumulation of terpenes by different yeast species [40]. It is known that some yeasts involved in the vinification process may exhibit β-glucosidase activity. This activity in *S. cerevisiae* has been proven in Riesling and Chardonnay musts and is limited due to the pH of the must and wine. The increase in the concentration of monoterpenes in wine may contribute significantly to the aroma profile of wines [39].

Sensory analysis revealed that the scores of the four sensory characteristics assessed for the 10 different wines were rather homogeneous (Table 4). The highest rated wines (highest total score) were obtained as a result of the application of mixed culture, in which *S. kudriavzevii* was sequentially inoculated on the sixth day of fermentation. The results of the study by Satora et al. [41] also confirmed that the presence of the DSM 3774 strain in mixed culture turned out to be beneficial for the quality of apple wines. Wines fermented with a pure culture of *S. kudriavzevii* DSM 3774 (formerly *S. bayanus*) and mixed culture of *S. cerevisiae* and *S. kudriavzevii* (in a ratio of 1:1) obtained the highest total scores during sensory analysis [41].

## 3. Materials and Methods

### 3.1. Microorganisms and the Preparation of Inoculate for Fermentation

*Saccharomyces cerevisiae* Johannisberg Riesling ŁOCK 105 and *Saccharomyces kudriavzevii* DSM 3774 were used for fermentations.

Yeast cultures were propagated at 28 °C on YEPD (Yeast Extract Peptone Dextrose) agar slants for 24 h, then transferred to 10 mL YPED of liquid medium and cultured for 24 h. Subsequently, the propagation in 190 mL liquid YPED medium was carried out for the next 24 h on a rotary shaker with a water bath at 120 rpm. The yeast cells were then harvested by centrifugation (10 min at 735× *g*) and washed twice with sterile water. The dry matter of the yeast was determined with the moisture analyzer. The yeast pellet was then suspended in a small amount of must and inoculated so that the yeast concentration in each trial was 0.5 g dry weight per liter. Mixed fermentation trials were performed by the simultaneous or sequential inoculation of *S. cerevisiae* (Sc) and *S. kudriavzevii* (Sk) with a different inoculum ratio (defined as the gram of Sc/Sk dry weight) of both tested strains as presented in Table 5.

### 3.2. Grape Must Composition and Fermentation

The Mondego Essential Medium Dry White concentrated juice was used for fermentation. Grape concentrate with an initial 70 °Blg extract was diluted 5 times to 17.5 °Blg, and then sweetened with sucrose up to 24 °Blg. The basic chemical parameters of the white grape must used for fermentations were as follows: extract 240.0 g/L; sucrose 94.1 g/L; reducing sugars 138.7 g/L; sugar-free extract 8.7 g/L; and titratable acidity 8.0 g/L. Before the fermentation process, the musts were pasteurized and inoculated with an appropriate pure or mixed culture.

Alcoholic fermentations were carried out for 28 days at a temperature of 20 °C in 0.5 L conical flasks. Each experiment (Table 5) was performed in triplicate. During fermentation, the weight loss of the samples connected with releasing CO_2_ was measured three times a week until the end of the process (a constant weight of two consecutive measurements). After fermentation, the young wines were separated from the sediment by carefully pouring them into other vessels (each repetition to one bottle) and kept for further clarification in the refrigerator for a week. Young, clarified wines were a subject of further analysis. All fermentation experiments were conducted in triplicate.

### 3.3. Enological Parameters Analysis

After fermentation, the concentration of ethanol, the total extract, the sugar-free extract, the reducing sugars, the titratable and volatile acidity were determined according to standard methods [42]. Titratable acidity was calculated from the volume of NaOH used for the titration (TitroLine Alpha, Schott Instruments GmbH (Mainz, Germany)) and expressed as g/L of tartaric acid. The reducing and total sugars were measured using the 3,5-dinitrosalicylic acid method [43]. The glycerol content was determined according to the standard method [42].

### 3.4. Solid Phase Microextraction–Gas Chromatography–Mass Spectrometry (SPME–GC–MS) Analysis of Volatile Aroma Components

Volatiles analysis was performed as described by Januszek and Satora [44]. Two mL of the wine sample was inserted into a 10-mL screw cap vial, suitable for volatile analysis. Subsequently, 1 g of NaCl and 0.1 mL of the internal standard (50 mg/L of 4-methyl-2-pentanol, 5 mg/L of ethyl nonanoate and 5 mg/L of anethol) were added. Three replicates per sample were prepared and analyzed.

The MPS autosampler (Gerstel, Mülheim an der Ruhr, Germany) with the functionality for automated SPME was used in the analyses. The equilibration time was 5 min at 40 °C. The volatile compounds of the head space were extracted and concentrated on a phase microextraction fiber coated with polydimethylsiloxane (100 μm PDMS, Supelco Inc., Bellefonte, PA, USA). The fiber was exposed to the sample headspace for 35 min at 40 °C. The volatile compounds adsorbed on the SPME fiber were desorbed at 250 °C (3 min) in the injector port of an Agilent Technologies 7890B chromatograph system (Agilent Technologies, Santa Clara, CA, USA) interfaced with a Pegasus HT TOFMS (Time-of-Flight Mass Spectrometry) detector (LECO Corporation, St. Joseph, MI, USA) operated in electron ionization mode. Chromatographic separation was performed on the Rtx-1ms capillary column (Crossbond 100% dimethyl polysiloxane, 30 m × 0.53 mm × 0.5 µm). The injector and detector temperature was 250 °C, while the separation of the compounds was initiated at 40 °C/3 min and then the temperature increased at an increment of 8 °C/min to 230 °C. Finally, the samples were held at the maximum temperature for 9 min. The carrier gas was helium at a constant flow rate of 1 mL/min held by an electronic pressure control. A transfer line and ion source temperature were set at 250 °C, and the ion source voltage was 70 eV. Analyte were transferred in the splitless mode. The mass spectrometer detector (MSD) was set to scan mode from *m*/*z* = 40 to *m*/*z* = 400.

Compounds were identified using mass spectral libraries and Linear Retention Indices, calculated based on a series of n-alkanes from C6 to C30. The qualitative and quantitative identification of volatile substances (showed in the Table 4; Sigma-Aldrich (Saint Louis, MO, USA)) was based on the comparison of retention times and peak surface area (based on the characteristic ion) read from sample and standard chromatograms. Other detected components (marked with superscript, Table 4) were determined semi-quantitatively (µg/L) from the ratio of the relative peak area of each identified component, to the relative peak area of the adequate internal standard (ethyl nonanoate for esters, anethol for terpenoids, and 4-methyl-2-pentanol for other components). Obtained results were analyzed using the National Institute of Standards and Technology (NIST) database [44].

### 3.5. Solid Phase Microextraction–Gas Chromatography–Flame Ionization (SPME-GC-FID) Analysis of Volatile Compounds

An analysis of selected volatiles was performed according to the method previously described by Januszek and Satora [44]. Each wine sample (2 mL) was transferred to a 15 mL vial, 2 mL of deionized water and 1 g of NaCl were added. The SPME device with PDMS fiber (100 μm, polydimethylsiloxane) was supplied by Supelco (Bellefonte, PA, USA). For sampling, the fiber was inserted into the headspace under magnetic stirring (300 RPM) for 35 min at 40 °C. The SPME device was then introduced into the injector port of a gas chromatograph (250 °C) and remained in the inlet for a time of 2 min. Determination of selected volatiles was performed on a Hewlett Packard 5890 Series II (Agilent Technologies, Santa Clara, CA, USA) chromatograph system with a flame ionization detector (FID). The volatile compounds were separated on an HP-INNOWax capillary column (30 m × 0.53 mm ID with 1.0 μm thickness, cross-linked polyethylene glycol stationary phase; Agilent, Santa Clara, CA, USA). The detector and injector temperature was set at 250 °C, and the column was heated using the following temperature program: 35 °C for 5 min at an increment of 5 °C/min to 110 °C, and then 40 °C/min to 220 °C and maintaining a constant temperature for 3 min. Helium was used as the carrier gas and the flow rate was set at 20 mL/min. Hydrogen was delivered at a flow speed of 33.0 mL/min, while for air it was 400 mL/min. The qualitative and quantitative identification of volatiles (acetaldehyde, amyl alcohols, ethyl acetate, isoamyl acetate, ethyl caproate, acetone, propanol, and isobutanol) was based on the comparison of retention times and the peak area read from sample and standard chromatograms. Quantitative calibration was performed using 4-methyl-2-pentanol as an internal standard. Each experiment was performed in triplicate.

### 3.6. Determination of Volatile Esters and Carbonyl Compounds

In addition to GC analysis, the volatile esters content was determined according to a standard method [30]. The analysis included saponification of the esters with sodium hydroxide solution and titration of excess NaOH with hydrochloric acid solution. The carbonyl compound content was determined using a method based on reaction with hydroxylamine hydrochloride and then titration of the resulting hydrochloric acid with a solution of 0.1 M NaOH in the presence of methyl orange [31].

### 3.7. Sensory Evaluation

Sensory evaluation was performed according to the Buxbaum method of positive rating [45,46]. The sensory panel consisted of 12 trained assessors (according to *EN ISO 8586:2014*) who evaluated the wine quality for a maximum of 20 points: color (0–2 points), aroma (0–4 points), taste (0–12 points), and clarity (0–2 points).

Sensory evaluation was performed in the tasting room. The wines were presented to the panelists in wine glasses that comply with the ISO standard, in insulated booths and with daylight illumination, the air temperature was 20 ± 1 °C. All wine samples were evaluated by the panel in random order of presentation. The white wine samples served were previously cooled to 10–12 °C. During three consecutive days, three sessions were held in which the panelist evaluated all individual wines on a daily basis. Each replicate presented on three consecutive days of tasting was poured from a separate bottle. Water was provided for mouth-rinsing between samples.

### 3.8. Data Statistical Analysis

The results were subjected to statistical interpretation. Mean values and standard deviations were calculated, and the significance of the variables was determined. Principal Component Analysis (PCA) was also performed to explore the correlation between the variables. IBM^®^ SPSS^®^ Statistics 19.0 (New York, NY, USA) was applied for statistical analysis of the results.

## 4. Conclusions

In conclusion, in most cases, the reasons for using mixed yeast cultures in winemaking are to improve the overall quality and complexity of the wine, modify its chemical profile (e.g., increase glycerol content, reduce acetic acid level, decrease ethanol yield), or, less frequently, control contaminating spoilage yeasts. The study presented in this article shows that no reduction in ethanol yield was found in the samples fermented with mixed culture, and even in some variants of the experiment, the ethanol content was higher. However, it can be concluded that the mixed cultures of *S. cerevisiae* and *S. kudriavzevii* used for fermentation increased the level of volatile compounds in white grape wines. The most promising results were obtained for mixed culture variants, in which *S. kudriavzevii* was sequentially inoculated on the sixth day of fermentation. These wines were rated the highest during sensory analysis and characterized by increased amounts of ethyl esters such as ethyl propanoate, ethyl 2-methylbutanoate, ethyl 2,4-hexadienoate, and ethyl hexadecanoate. Differences related to the production of ethanol, terpenes, and other volatile compounds were noticeable, especially when *S. kudriavzevii* was introduced on the sixth day of fermentation with *S. cerevisiae*, regardless of the proportion of the two yeast strains used. It is possible that yeast cells of *S. kudriavzevii* under stress conditions (including the presence of ethanol) were lysed and became a source of nitrogen and other nutrients for *S. cerevisiae* cells that previously dominated the fermentation environment. It is also possible that under these conditions there was an increased production of enzymes involved in the formation of volatile compounds, especially secondary metabolites, but this would require elucidation and confirmation in further studies.

## Figures and Tables

**Table 1:** The principal oenological parameters of wines obtained using monocultures or mixed cultures of *Saccharomyces cerevisiae* (Sc) and *S. kudriavzevii* (Sk).

Strains	Ethanol [%vol.]	Total Extract	Total Sugars	Sucrose	Reducing Sugars	Sugar-Free Extract	Glycerol	Titratable Acidity	Volatile Acidity
		[g/L]							
Sc	9.88 ± 0.14 ^c^	58.2 ± 2.4 ^f^	27.1 ± 0.2 ^f^	8.1 ± 0.1 ^e^	19.1 ± 0.2 ^d^	31.1 ± 2.6 ^d^	8.1 ± 0.1 ^d^	8.9 ± 0.1 ^b^	0.22 ± 0.00 ^b^
Sk	8.69 ± 0.08 ^a^	69.8 ± 0.2 ^h^	36.0 ± 0.2 ^h^	13.9 ± 0.1 ^g^	22.2 ± 0.2 ^f^	33.8 ± 0.2 ^e^	7.7 ± 0.1 ^c^	10.1 ± 0.1 ^c^	0.32 ± 0.01 ^d^
Sc + Sk (3:2)	8.99 ± 0.12 ^b^	65.0 ± 0.0 ^g^	32.2 ± 0.8 ^g^	10.9 ± 0.6 ^f^	21.2 ± 0.4 ^e^	32.8 ± 0.8 ^de^	7.7 ± 0.2 ^c^	8.4 ± 0.2 ^a^	0.23 ± 0.02 ^b^
Sc + Sk_1_ (3:2)	10.06 ± 0.05 ^c^	44.6 ± 0.5 ^c^	16.7 ± 0.1 ^c^	0.7 ± 0.0 ^a^	16.0 ± 0.2 ^c^	27.8 ± 0.5 ^c^	7.4 ± 0.1 ^b^	9.0 ± 0.1 ^b^	0.19 ± 0.01 ^a^
Sc + Sk_3_ (3:2)	10.51 ± 0.05 ^d^	39.7 ± 0.2 ^b^	15.5 ± 0.7 ^b^	1.9 ± 0.7 ^b^	13.6 ± 0.2 ^b^	24.2 ± 0.8 ^b^	7.4 ± 0.1 ^b^	8.4 ± 0.1 ^a^	0.23 ± 0.01 ^b^
Sc + Sk_6_ (3:2)	11.18 ± 0.05 ^e^	29.8 ± 0.2 ^a^	12.2 ± 0.2 ^a^	0.0 ± 0.0 ^a^	12.2 ± 0.2 ^a^	17.7 ± 0.1 ^a^	7.5 ± 0.2 ^b,c^	8.6 ± 0.2 ^a^	0.18 ± 0.01 ^a^
Sc + Sk (99:1)	9.91 ± 0.14 ^c^	54.5 ± 0.6 ^e^	25.6 ± 0.2 ^e^	6.6 ± 0.1 ^d^	19.1 ± 0.3 ^d^	28.9 ± 0.5 ^c^	8.1 ± 0.1 ^d^	8.6 ± 0.3 ^a,b^	0.28 ± 0.01 ^c^
Sc + Sk_1_ (99:1)	10.02 ± 0.05 ^c^	49.4 ± 0.4 ^d^	20.9 ± 0.4 ^d^	5.1 ± 0.1 ^c^	15.9 ± 0.2 ^c^	28.4 ± 0.5 ^c^	7.4 ± 0.1 ^b^	8.9 ± 0.1 ^b^	0.18 ± 0.01 ^a^
Sc + Sk_3_ (99:1)	10.42 ± 0.05 ^d^	40.0 ± 0.5 ^b^	17.3 ± 0.7 ^c^	1.9 ± 0.7 ^b^	15.4 ± 0.5 ^c^	22.7 ± 0.7 ^b^	6.4 ± 0.1 ^a^	8.4 ± 0.2 ^a^	0.23 ± 0.01 ^b^
Sc + Sk_6_ (99:1)	11.19 ± 0.06 ^e^	29.6 ± 0.6 ^a^	11.9 ± 0.6 ^a^	0.0 ± 0.0 ^a^	11.9 ± 0.3 ^a^	17.7 ± 0.1 ^a^	7.6 ± 0.0 ^c^	8.5 ± 0.1 ^a^	0.22 ± 0.01 ^b^
Sig. ^1^	***	***	***	***	***	***	***	***	**

The mean values with different letters (a–h) in the same column are significantly different (*p* < 0.05); “±” indicates standard deviation. ^1^ Sig.: significance; **, ***—display the significance at 5, 1 and 0.5% by least significant difference.

**Table 2:** Results of the GC-FID analysis of main volatile aroma compounds of wines obtained using monocultures or mixed cultures of *Saccharomyces cerevisiae* (Sc) and *S. kudriavzevii* (Sk).

Strains	Ethyl Acetate	Isoamyl Acetate	Ethyl Caproate	Volatile Esters ^2^	Acetone	Acetaldehyde	Carbonyl Compounds ^2^	Propanol	Isobutanol	Amyl Alcohols
	[mg/L]									
Sc	76.2 ±0.4 ^a,b^	2.7 ± 0.2 ^a^	0.7 ± 0.2 ^a^	316.8 ± 0.0 ^d^	7.2 ± 2.9 ^a,b^	13.6 ± 1.1 ^a^	44.0 ± 0.1 ^e^	60.2 ± 4.0 ^a,c^	38.3 ± 2.2 ^c^	68.8 ± 1.8 ^c^
Sk	73.9 ± 1.6 ^a^	2.4 ± 0.2 ^a^	0.5 ± 0.4 ^a^	299.2 ± 0.0 ^c^	8.0 ± 3.3 ^a,b^	16.7 ± 2.3 ^b,c^	99.0 ±0.1 ^g^	48.5 ± 5.0 ^b^	36.9 ± 2.8 ^b,c^	68.8 ± 0.9 ^c^
Sc + Sk (3:2)	73.9 ± 5.4 ^a^	2.4 ± 0.1 ^a^	0.5 ± 0.0 ^a^	305.1 ± 8.3 ^c^	10.8 ±0.5 ^b^	15.4 ± 2.7 ^b,c^	25.7 ± 5.2 ^c^	59.5 ± 3.5 ^a,d^	37.4 ± 0.9 ^b,c^	67.8 ± 0.7 ^b,c^
Sc + Sk_1_ (3:2)	84.5 ± 5.1 ^c,d^	2.4 ± 0.0 ^a^	0.7 ± 0.2 ^a^	299.2 ± 0.0 ^c^	3.9 ± 0.9 ^a^	14.3 ± 1.9 ^a,b^	22.0 ± 0.1 ^b,c^	53.4 ± 0.1 ^b,d^	31.4 ± 3.9 ^a^	63.6 ± 1.4 ^a^
Sc + Sk_3_ (3:2)	81.3 ± 2.9 ^b,c^	2.6 ± 0.1 ^a^	0.9 ± 0.5 ^a^	316.8 ± 0.0 ^d^	6.1 ± 0.4 ^a,b^	13.3 ± 0.9 ^a^	33.0 ± 0.1 ^d^	56.9 ± 2.0 ^a^	36.1 ± 1.0 ^a,b^	65.7 ± 0.7 ^a,b^
Sc + Sk_6_ (3:2)	99.0 ± 1.5 ^e^	2.7 ± 0.1 ^a^	0.7 ± 0.1 ^a^	264.0 ± 0.0 ^a^	4.7 ± 0.3 ^a^	23.1 ± 0.6 ^d^	55.0 ± 0.1 ^f^	66.1 ± 3.5 ^c^	40.4 ± 1.7 ^b,c^	67.9 ± 0.4 ^b,c^
Sc + Sk (99:1)	69.5 ± 2.9 ^a^	2.4 ± 0.1 ^a^	0.5 ± 0.0 ^a^	264.0 ± 0.0 ^a^	9.9 ± 1.6 ^b^	14.2 ± 0.9 ^a,b^	22.0 ± 0.1 ^b,c^	59.8 ± 4.5 ^a^	34.6 ± 4.1 ^a,b^	70.0 ± 1.6 ^c^
Sc + Sk_1_ (99:1)	83.0 ± 3.8 ^c,d^	2.5 ± 0.1 ^a^	0.5 ± 0.0 ^a^	299.2 ± 0.0 ^c^	4.7 ± 0.6 ^a^	16.0 ± 1.2 ^b,c^	18.3 ± 5.2 ^b^	59.5 ± 2.8 ^a,d^	33.1 ± 3.1 ^a,c^	64.4 ± 0.8 ^a^
Sc + Sk_3_ (99:1)	90.1 ± 4.6 ^d^	2.6 ± 0.1 ^a^	0.5 ± 0.0 ^a^	287.5 ± 8.3 ^b^	4.1 ± 0.7 ^a^	15.9 ± 1.4 ^b,c^	11.0 ± 0.1 ^a^	59.7 ± 2.0 ^a^	37.7 ± 3.1 ^b,c^	65.6 ± 0.4 ^a,b^
Sc + Sk_6_ (99:1)	89.6 ± 3.4 ^d^	2.5 ± 0.1 ^a^	0.5 ± 0.0 ^a^	264.0 ± 0.0 ^a^	7.7 ± 1.1 ^a,b^	17.7 ± 0.8 ^c^	44.0 ± 0.1 ^e^	63.1 ± 1.9 ^a,c^	37.5 ± 0.1 ^b,c^	67.4 ± 0.4 ^b,c^
Sig. ^1^	**	ns	ns	***	*	***	***	**	**	**

The mean values with different letters (a–g) in the same column are significantly different (*p* < 0.05); “±” indicates standard deviation. ^1^ Sig.: significance; *, **, ***—display the significance at 5, 1 and 0.5% by least significant difference; ns: not significant; ^2^—Compound concentrations determined according to standard methods [30,31].

**Table 3:** A heat map of 89 volatile components [μg/L] produced by pure and mixed cultures of *S. cerevisiae* (Sc) and *S. kudriavzevii* (Sk). The highest concentration of a specific compound in a row is marked in dark green and the lowest content is marked in dark red.

	Code	LRI ^2^	Sc	Sk	Sc + Sk (3:2)	Sc + Sk_1_ (3:2)	Sc + Sk_3_ (3:2)	Sc + Sk_6_ (3:2)	Sc + Sk (99:1)	Sc + Sk_1_ (99:1)	Sc + Sk_3_ (99:1)	Sc + Sk_6_ (99:1)	Sig. ^1^
Ethyl esters													
Ethyl propanoate	**E1**	699	0.0 ^a^	0.0 ^a^	13.9 ^a,b^	25.4 ^b,c^	12.4 ^a,b^	34.7 ^c^	25.0 ^b,c^	7.6 ^a,b^	13.6 ^a,b^	44.0 ^c^	***
Ethyl pyruvate ^3^	**E2**	785	0.0 ^a^	0.0 ^a^	32.9 ^a–c^	26.4 ^a–c^	2.9 ^a^	34.7 ^a–c^	68.8 ^b,c^	15.6 ^a,b^	81.3 ^c^	13.2 ^a,b^	*
Ethyl butanoate	**E3**	789	29.7 ^a^	6.7 ^a^	55.7 ^a^	23.2 ^a^	7.8 ^a^	74.3 ^a,b^	162.9 ^b^	19.5 ^a^	40.1 ^a^	112.2 ^a,b^	*
Ethyl lactate	**E4**	798	4.2	79.7	19.8	26.4	0.9	6.0	9.2	29.5	5.4	62.0	ns
Ethyl (Z)-2-butenoate ^3^	**E5**	830	0.0 ^a^	0.0 ^a^	0.0 ^a^	0.0 ^a^	0.0 ^a^	1.2 ^a,b^	0.0 ^a^	0.0 ^a^	0.0 ^a^	1.8 ^b^	**
Ethyl 2-methylbutanoate ^3^	**E6**	847	0.0 ^a^	0.0 ^a^	0.0 ^a^	0.0 ^a^	0.0 ^a^	3.7 ^b^	0.0 ^a^	0.0 ^a^	0.0 ^a^	3.7 ^b^	***
Ethyl 3-hydroxybutyrate ^3^	**E7**	949	0.0 ^a^	6.9 ^c,d^	0.0 ^a^	0.0 ^a^	0.0 ^a^	8.9 ^d^	5.2 ^b–d^	5.7 ^b–d^	3.0 ^a–c^	1.8 ^b^	***
Ethyl 2,4-hexadienoate ^3^	**E8**	1089	0.0 ^a^	0.0 ^a^	0.0 ^a^	0.0 ^a^	0.0 ^a^	57.6 ^b^	0.0 ^a^	0.0 ^a^	0.0 ^a^	72.3 ^b^	***
Ethyl heptanoate ^3^	**E9**	1095	2.9 ^a^	1.0 ^a^	5.1 ^a^	0.0 ^a^	0.0 ^a^	11.7 ^a^	13.8 ^a^	12.8 ^a^	0.0 ^a^	36.0 ^b^	***
Ethyl octanoate	**E10**	1180	77 ^a^	23 ^a^	1636 ^b^	1754 ^b^	41 ^a^	1699 ^b^	3463 ^c^	1074 ^b^	166 ^a^	1994 ^b,c^	***
Ethyl 9-decenoate ^3^	**E11**	1389	0.29 ^a^	0.00 ^a^	5.55 ^c^	1.63 ^a^	0.36 ^a^	9.71 ^d^	4.85 ^b,c^	6.14 ^c^	1.54 ^a^	3.78 ^b^	***
Ethyl decanoate	**E12**	1397	9.6 ^a,b^	3.9 ^a^	115.6 ^b,c^	304.8 ^e^	18.2 ^a,b^	183.6 ^c,d^	306.8 ^e^	250.1 ^d,e^	10.4 ^a,b^	272.6 ^d,e^	***
Ethyl 3-hydroxydecanoate ^3^	**E13**	1539	0.00 ^a^	0.00 ^a^	1.38 ^c,d^	1.68 ^d^	2.24 ^e^	1.46 ^c,d^	0.70 ^b^	1.44 ^c,d^	1.58 ^d^	0.99 ^b,c^	***
Ethyl dodecanoate	**E14**	1581	1.8 ^a,b^	0.8 ^a^	9.8 ^b–e^	17.2 ^e^	3.1 ^a–c^	10.6 ^c–e^	10.6 ^c–e^	8.3 ^a–d^	7.4 ^a–d^	12.2 ^d,e^	**
Ethyl 3-hydroxydodecanoate ^3^	**E15**	1743	0.09 ^a,b^	0.90 ^e^	0.18 ^a,b^	0.54 ^c^	0.27 ^a–c^	0.58 ^c,d^	0.00 ^a^	0.10 ^a^	0.34 ^b,c^	0.87 ^d,e^	***
Ethyl tetradecanoate	**E16**	1790	1.17	0.95	6.18	2.75	1.34	2.75	2.36	1.28	4.55	2.86	ns
Ethyl pentadecanoate ^3^	**E17**	1880	0.18	0.35	1.93	0.32	0.27	0.52	0.18	0.60	1.64	0.89	ns
Ethyl E-11-hexadecenoate ^3^	**E18**	1974	0.45	0.00	1.19	0.49	0.00	2.24	2.39	0.00	3.24	0.98	ns
Ethyl hexadecanoate	**E19**	1990	4.5 ^a,b^	7.4 ^b,c^	6.8 ^a,b^	4.5 ^a,b^	2.4 ^a,b^	12.5 ^c,d^	7.0 ^a,b^	1.5 ^a^	13.2 ^d^	14.9 ^d^	***
Ethyl octadecanoate	**E20**	2189	0.41 ^a,b^	1.02 ^b,c^	0.10 ^a^	0.49 ^a,b^	0.12 ^a^	1.08 ^b,c^	0.22 ^a,b^	0.85 ^a–c^	0.83 ^a–c^	1.42 ^c^	*
Acetates													
Isobutyl acetate	**E21**	756	5.3 ^a,b^	0.0 ^a^	9.6 ^a,b^	1.4 ^a,b^	1.4 ^a,b^	8.5 ^a,b^	29.9 ^c^	7.9 ^a,b^	1.8 ^a,b^	16.9 ^b,c^	*
Butyl acetate	**E22**	805	0.0 ^a^	0.0 ^a^	3.1 ^a^	1.5 ^a^	0.0 ^a^	0.0 ^a^	11.7 ^c^	0.0 ^a^	0.0 ^a^	6.9 ^b^	***
Hexyl acetate	**E23**	1008	15.2 ^b^	5.4 ^a–c^	6.5 ^a,b^	2.6 ^a,b^	1.7 ^a^	3.7 ^a,b^	15.2 ^b,c^	0.0 ^a^	0.0 ^a^	22.4 ^c^	**
2-Phenylethyl acetate	**E24**	1228	4.1 ^a^	3.8 ^a^	12.7 ^a,b^	61.4 ^e^	13.7 ^a,b^	27.2 ^b,c^	31.8 ^c,d^	46.3 ^d,e^	35.8 ^c,d^	33.2 ^c,d^	***
Other esters													
2-Methylbutyl butanoate	**E25**	1020	8.3 ^b^	0.9 ^a^	1.1 ^a^	1.5 ^a^	0.0 ^a^	0.7 ^a^	6.8 ^b^	0.0 ^a^	0.0 ^a^	6.0 ^b^	***
Methyl octanoate	**E26**	1126	0.0 ^a^	0.0 ^a^	4.6 ^a^	34.7 ^b^	4.3 ^a^	3.7 ^a^	0.0 ^a^	2.9 ^a^	0.0 ^a^	3.6 ^a^	***
Diethyl succinate	**E27**	1153	14.9 ^a^	19.9 ^a^	33.2 ^a–c^	31.6 ^a–c^	18.3 ^a^	52.6 ^c,d^	37.6 ^a–c^	73.6 ^d^	49.8 ^b–d^	22.2 ^a,b^	***
Methyl decanoate	**E28**	1324	0.0 ^a^	0.0 ^a^	0.0 ^a^	6.3 ^b^	1.1 ^a^	0.5 ^a^	0.0 ^a^	5.5 ^b^	0.2 ^a^	0.9 ^a^	***
Ethyl 3-methylbutyl succinate ^3^	**E29**	1430	0.00 ^a^	0.00 ^a^	1.37 ^b^	0.50 ^b,c^	0.07 ^a,b^	0.56 ^c^	0.64 ^c^	0.49 ^b,c^	0.34 ^a–c^	0.47 ^a–c^	***
3-Methylbutyl octanoate ^3^	**E30**	1450	0.00 ^a^	0.00 ^a^	0.00 ^a^	1.30 ^b^	0.72 ^a,b^	0.60 ^a,b^	0.00 ^a^	1.34 ^b^	0.42 ^a,b^	0.66 ^a,b^	*
1-methylethyl dodecanoate ^3^	**E31**	1614	0.35	0.50	0.32	1.56	2.30	1.01	0.32	1.49	1.15	0.76	ns
Benzyl benzoate	**E32**	1755	1.24	1.51	2.15	1.65	2.17	1.64	1.13	1.49	2.54	0.64	ns
Methyl 15-methylhexadecanoate ^3^	**E33**	1970	0.51	0.62	0.36	0.49	0.34	1.43	0.29	1.18	0.50	0.59	ns
**Alcohols and polyols**													
2,3-Butanediol	**A1**	770	712 ^a–c^	1434 ^d^	226 ^a^	568 ^a–c^	693 ^a–c^	1482 ^d^	1072 ^b–d^	496 ^a–c^	436 ^a,b^	1177 ^c,d^	***
3-Hexanol ^3^	**A2**	784	14.5 ^b,c^	11.8 ^a–c^	23.4 ^c^	7.1 ^a,b^	2.2 ^a,b^	3.9 ^a,b^	8.9 ^a,b^	9.8 ^a,b^	1.4 ^a^	0.9 ^a^	*
1-Hexanol	**A3**	865	13.2 ^c^	14.9 ^c^	26.0 ^d^	11.8 ^b,c^	4.4 ^a,b^	3.7 ^a^	27.2 ^d^	10.0 ^a–c^	7.1 ^a–c^	10.2 ^a–c^	***
1-Heptanol ^3^	**A4**	971	0.00 ^a^	0.00 ^a^	8.62 ^b^	7.98 ^b^	3.60 ^a,b^	1.34 ^a^	5.39 ^a,b^	5.42 ^a,b^	3.18 ^a,b^	0.00 ^a^	*
2-Ethyl-1-hexanol	**A5**	1034	27.8 ^a–c^	20.6 ^a,b^	65.0 ^d,e^	43.0 ^b–d^	23.4 ^a,b^	9.5 ^a^	54.2 ^c–e^	61.3 ^d,e^	25.7 ^a–c^	74.6 ^e^	***
1-Octanol	**A6**	1068	5.18 ^b,c^	2.66 ^a^	2.38 ^a^	5.33 ^b,c^	5.56 ^b,c^	2.88 ^a^	3.76 ^a,b^	6.52 ^c^	4.83 ^b,c^	3.84 ^a,b^	***
Phenylethyl alcohol	**A7**	1114	710 ^d^	189 ^a,b^	388 ^b,c^	2016 ^f^	553 ^c,d^	272 ^a,b^	135 ^a^	964 ^e^	682 ^d^	224 ^a,b^	***
1-Nonanol	**A8**	1156	1.95 ^a,b^	7.91 ^d^	4.68 ^b,c^	7.50 ^dc^	9.10 ^d^	6.48 ^c,d^	2.89 ^a,b^	4.68 ^b,c^	8.17 ^d^	0.00 ^a^	***
1-Decanol	**A9**	1272	0.00 ^a^	0.00 ^a^	0.00 ^a^	0.00 ^a^	7.90 ^e^	3.50 ^d^	0.00 ^a^	0.00 ^a^	0.56 ^b^	1.60 ^c^	***
1-Undecanol ^3^	**A10**	1374	0.49	6.21	3.67	0.00	0.00	4.66	1.50	0.00	0.12	1.51	ns
1-Dodecanol	**A11**	1480	2.01 ^a^	1.99 ^a^	3.56 ^a,b^	3.88 ^a,b^	3.43 ^a,b^	2.86 ^a^	1.47 ^a^	2.54 ^a^	5.64 ^b^	2.62 ^a^	*
1-Tridecanol ^3^	**A12**	1577	0.68	0.78	0.93	1.23	0.99	0.45	1.02	0.85	1.23	0.46	ns
1-Tetradecanol	**A13**	1661	1.20 ^a,b^	1.55 ^b^	0.71 ^a,b^	3.50 ^c^	0.78 ^a,b^	0.93 ^a,b^	0.82 ^a,b^	0.34 ^a^	1.08 ^a,b^	1.11 ^a,b^	***
1-Pentadecanol ^3^	**A14**	1787	0.36	0.77	0.29	0.13	0.06	0.16	0.00	0.37	0.29	0.45	ns
1-Hexadecanol	**A15**	1877	0.56 ^a,b^	1.68 ^b^	0.22 ^a^	1.39 ^a,b^	0.73 ^a,b^	1.51 ^b^	0.27 ^a^	0.70 ^a,b^	0.62 ^a,b^	2.78 ^c^	***
1-Octadecanol	**A16**	2075	0.22 ^a,b^	0.44 ^b,c^	0.00 ^a^	0.00 ^a^	0.14 ^a,b^	0.09 ^a^	0.00 ^a^	0.00 ^a^	0.00 ^a^	0.66 ^c^	***
Volatile acids													
Hexanoic acid	**VA1**	982	67.5 ^d,e^	38.9 ^a–c^	66.5 ^c–e^	46.8 ^a–d^	62.2 ^c–e^	98.0 ^f^	28.1 ^a^	60.7 ^b–e^	33.8 ^a,b^	77.2 ^e,f^	***
Heptanoic acid ^3^	**VA2**	1080	4.3 ^a^	7.6 ^a–c^	5.7 ^a^	11.6 ^b–d^	7.3 ^a,b^	15.0 ^d^	6.4 ^a^	12.0 ^c,d^	7.7 ^a–c^	14.4 ^d^	***
Octanoic acid	**VA3**	1160	116 ^a^	108 ^a^	134 ^a,b^	480 ^e^	370 ^d,e^	255 ^b–d^	144 ^a,b^	450 ^e^	274 ^c,d^	233 ^a–c^	***
n-Decanoic acid	**VA4**	1368	21.9 ^a,b^	23.1 ^a,b^	33.3 ^b,c^	74.2 ^e^	73.2 ^e^	41.8 ^b,c^	8.8 ^a^	68.1 ^d,e^	48.6 ^c,d^	39.0 ^b,c^	***
Dodecanoic acid	**VA5**	1554	2.44	3.82	1.68	5.43	4.62	3.34	1.11	3.37	3.72	4.08	ns
n-Hexadecanoic acid	**VA6**	1965	1.18 ^a–c^	2.69 ^c,d^	0.32 ^a,b^	0.00 ^a^	0.27 ^a,b^	2.02 ^b,c^	0.00 ^a^	0.00 ^a^	0.07 ^a^	3.95 ^d^	***
Carbonyl compounds													
Hexanal	**CC1**	778	11.8 ^b^	8.4 ^b^	10.6 ^b^	9.2 ^b^	0.9 ^a^	2.5 ^a^	21.8 ^d^	11.4 ^b^	3.4 ^a^	17.1 ^c^	***
Butyrolactone ^3^	**CC2**	915	21.6	12.6	13.1	35.2	25.2	36.7	32.4	29.8	28.2	19.2	ns
Benzaldehyde	**CC3**	959	7.6 ^c,d^	6.2 ^b,c^	6.6 ^b,c^	15.1 ^e^	8.1 ^c,d^	3.0 ^a,b^	8.7 ^c,d^	8.8 ^c,d^	11.0 ^d^	0.0 ^a^	***
6-Methyl-5-hepten-2-one ^3^	**CC4**	975	0.0 ^a^	0.0 ^a^	5.3 ^a^	0.0 ^a^	0.0 ^a^	0.0 ^a^	30.1 ^b^	6.2 ^a^	4.1 ^a^	1.1 ^a^	***
Acetophenone	**CC5**	1053	3.55 ^b–d^	2.50 ^b^	0.00 ^a^	5.05 ^d^	2.83 ^b,c^	2.57 ^b^	5.37 ^d^	3.89 ^b–d^	4.63 ^c,d^	0.00 ^a^	***
Nonanal	**CC6**	1102	18.8 ^b^	0.0 ^a^	3.9 ^a^	10.3 ^a,b^	10.5 ^a,b^	11.2 ^a,b^	1.1 ^a^	113.3 ^d^	0.7 ^a^	35.2 ^c^	***
Decanal	**CC7**	1183	17.1 ^a^	6.4 ^a^	68.4 ^b^	13.9 ^a^	17.2 ^a^	12.0 ^a^	8.4 ^a^	11.1 ^a^	12.5 ^a^	3.7 ^a^	***
Dodecanal	**CC8**	1407	3.00 ^d^	0.46 ^a^	9.02 ^e^	2.35 ^b–d^	1.14 ^a–c^	0.98 ^a–c^	3.44 ^d^	2.76 ^c,d^	2.29 ^b–d^	0.87 ^a,b^	***
Benzophenone	**CC9**	1603	2.72 ^b–e^	2.15 ^b,c^	3.15 ^c–f^	3.45 ^d–f^	3.85 ^e,f^	0.60 ^a^	4.20 ^f^	1.76 ^b^	2.44 ^b–d^	0.06 ^a^	***
Tetradecanal	**CC10**	1611	1.83 ^a^	0.49 ^a^	4.70 ^b^	1.27 ^a^	0.74 ^a^	0.55 ^a^	1.39 ^a^	2.55 ^a^	0.70 ^a^	0.86 ^a^	**
Monoterpenes													
p-Cymene	**T1**	1027	0.0 ^a^	0.0 ^a^	30.0 ^b^	70.4 ^c,d^	51.7 ^b,c^	32.9 ^b^	83.1 ^d^	63.7 ^c,d^	66.0 ^c,d^	27.1 ^b^	***
α-Terpinene	**T2**	1030	0.00 ^a^	0.00 ^a^	6.85 ^f^	1.15 ^a–c^	1.06 ^a–c^	0.96 ^a,b^	3.48 ^d,e^	2.26 ^b–d^	4.00 ^e^	2.67 ^c–e^	***
α-Ocimene ^3^	**T3**	1058	0.0 ^a^	0.0 ^a^	11.9 ^b^	2.2 ^a^	1.0 ^a^	0.0 ^a^	9.7 ^b^	1.8 ^a^	0.0 ^a^	2.3 ^a^	***
γ-Terpinene ^3^	**T4**	1060	0.00 ^a^	0.00 ^a^	8.38 ^d^	6.63 ^c,d^	4.37 ^b,c^	4.01 ^b^	6.64 ^c,d^	4.19 ^b,c^	3.55 ^b^	3.94 ^b^	***
Dihydromyrcenol ^3^	**T5**	1076	9.4 ^b^	7.4 ^b^	18.8 ^d^	14.6 ^c^	10.8 ^b^	3.8 ^a^	23.1 ^e^	9.2 ^b^	8.7 ^b^	4.3 ^a^	***
α-Terpinolene	**T6**	1093	5.8 ^a,b^	3.8 ^a^	10.8 ^c^	5.4 ^a,b^	4.5 ^a^	6.6 ^a,b^	10.4 ^c^	5.0 ^a^	4.1 ^a^	8.2 ^b,c^	***
Linolool	**T7**	1106	61 ^a,b^	50 ^a,b^	110 ^b,c^	12 ^a^	4 ^a^	51 ^a,b^	175 ^c^	8 ^a^	16 ^a^	61 ^a,b^	***
Myrcenol ^3^	**T8**	1118	0.00 ^a^	0.00 ^a^	0.00 ^a^	5.03 ^c^	1.92 ^a,b^	6.89 ^d^	0.00 ^a^	3.89 ^c^	3.66 ^b,c^	7.22 ^d^	***
Camphore	**T9**	1139	7.5 ^b^	1.6 ^a^	8.6 ^b,c^	14.9 ^d^	8.6 ^b,c^	5.4 ^a,b^	13.4 ^c,d^	14.1 ^d^	13.7 ^c,d^	7.8 ^b^	***
Ocimenol ^3^	**T10**	1149	1.40 ^a,b^	2.55 ^a–c^	2.02 ^a,b^	5.91 ^c^	3.44 ^a–c^	12.87 ^d^	0.92 ^a^	6.12 ^c^	4.93 ^b,c^	11.25 ^d^	***
Dihydro-γ-terpineol ^3^	**T11**	1158	7.86 ^a–c^	6.08 ^a,b^	9.64 ^a–c^	27.20 ^d^	19.49 ^c,d^	3.13 ^a,b^	9.27 ^a–c^	13.04 ^b,c^	5.17 ^a,b^	0.00 ^a^	***
Terpinen-4-ol	**T12**	1163	3.1 ^a,b^	3.9 ^a,b^	4.7 ^a–c^	9.8 ^d^	0.5 ^a^	15.2 ^e^	7.8 ^b–d^	9.4 ^c,d^	1.3 ^a^	16.4 ^e^	***
α-Terpineol	**T13**	1171	126 ^a^	248 ^a^	229 ^a^	346 ^a^	193 ^a^	699 ^b^	260 ^a^	345 ^a^	263 ^a^	728 ^b^	***
α-Terpinyl acetate ^3^	**T14**	1350	2.14 ^d–f^	3.17 ^f^	1.93 ^c–f^	0.11 ^a^	0.08 ^a^	1.69 ^c–e^	1.48 ^b–d^	0.20 ^a,b^	0.73 ^a–c^	2.90 ^e,f^	***
β-Damascenone	**T15**	1384	0.00 ^a^	0.00 ^a^	0.00 ^a^	0.00 ^a^	0.00 ^a^	0.67 ^c^	0.00 ^a^	0.00 ^a^	0.00 ^a^	0.50 ^b^	***
Geranyl acetate ^3^	**T16**	1392	1.62 ^a^	6.17 ^b^	3.05 ^a^	0.63 ^a^	0.52 ^a^	1.63 ^a^	2.89 ^a^	0.24 ^a^	0.35 ^a^	1.67 ^a^	***
Geranyl acetone ^3^	**T17**	1446	2.60 ^a,b^	1.61 ^a^	2.71 ^a,b^	5.45 ^d^	4.49 ^c,d^	2.31 ^a,b^	3.12 ^b,c^	7.18 ^e^	2.14 ^a,b^	3.34 ^b,c^	***
**Sesquiterpenes**													
β-Farnesene	**T18**	1460	0.00 ^a^	0.00 ^a^	0.00 ^a^	0.00 ^a^	0.53 ^b^	0.56 ^b^	0.00 ^a^	0.00 ^a^	0.80 ^c^	0.39 ^b^	***
Nerolidol ^3^	**T19**	1575	0.77 ^a^	1.48 ^a–c^	2.33 ^b–d^	1.30 ^a,b^	0.79 ^a^	2.59 ^c,d^	2.04 ^b,c^	1.50 ^a–c^	1.73 ^a–c^	3.37 ^d^	***
2,3-Dihydrofarnesol ^3^	**T20**	1696	0.64 ^a^	2.21 ^a^	1.38 ^a^	1.96 ^a^	1.27 ^a^	6.42 ^b,c^	0.70 ^a^	1.30 ^a^	3.11 ^a,b^	8.83 ^c^	***
Farnesol ^3^	**T21**	1718	1.85 ^a^	16.38 ^d^	3.67 ^a,b^	1.78 ^a^	1.44 ^a^	6.23 ^b^	1.29 ^a^	0.76 ^a^	1.78 ^a^	9.97 ^c^	***
**Other**													
Benzothiazole	**O1**	1186	1.01 ^a^	1.85 ^a^	3.26 ^a^	9.40 ^b^	1.69 ^a^	1.66 ^a^	3.75 ^a^	4.76 ^a^	2.47 ^a^	0.77 ^a^	***
Octane, 1,1’-oxybis- ^3^	**O2**	1657	0.96 ^a,b^	0.00 ^a^	1.43 ^a,b^	1.09 ^a,b^	2.31 ^b,c^	0.55 ^a,b^	1.14 ^a,b^	3.94 ^c^	1.47 ^a,b^	1.05 ^a,b^	*
Phenanthrene ^3^	**O3**	1778	0.26 ^b,c^	0.19 ^a,b^	0.00 ^a^	0.50 ^d^	0.45 ^c,d^	0.28 ^b,c^	0.00 ^a^	0.53 ^d^	0.63 ^d^	0.06 ^a^	***

^1^ Sig.: significance; *, **, ***—display the significance at 5, 1 and 0.5% by least significant difference; ns: not significant. Values with different superscript roman letters (a–f) in the same row are significantly different according to the Duncan test (*p* < 0.05). ^2^ LRI—Linear Retention Index. ^3^ Determined semi-quantitatively by measuring the relative peak area of each identified compound, according to the NIST database, in relation to that of the internal standard.

**Table 4:** The results of sensory analysis of wines obtained using monocultures or mixed cultures of *Saccharomyces cerevisiae* (Sc) and *S. kudriavzevii* (Sk).

Strains	Color	Aroma	Taste	Clearness	Total
Sc	1.2 ± 0.5 ^a^	2.3 ± 0.8 ^a^	7.2 ± 2.4 ^a,b^	1.1 ± 0.3 ^a^	11.8 ± 3.4 ^a,b^
Sk	1.4 ± 0.4 ^a,b^	2.3 ± 0.8 ^a^	7.8 ± 1.8 ^a,b^	1.3 ± 0.4 ^a,b^	12.8 ± 2.4 ^a,b^
Sc + Sk (3:2)	1.3 ± 0.5 ^a,b^	1.7 ± 1.0 ^a^	6.7 ± 1.0 ^a^	1.4 ± 0.4 ^a,b^	11.0 ± 2.2 ^a^
Sc + Sk_1_ (3:2)	1.6 ± 0.5 ^a,b^	2.2 ± 0.7 ^a^	8.0 ± 1.7 ^a,b^	1.7 ± 0.4 ^b^	13.5 ± 2.5 ^a,b^
Sc + Sk_3_ (3:2)	1.5 ± 0.4 ^a,b^	2.5 ± 0.6 ^a^	7.2 ± 1.1 ^a,b^	1.4 ± 0.5 ^a,b^	12.6 ± 1.2 ^a,b^
Sc + Sk_6_ (3:2)	1.7 ± 0.4 ^a,b^	2.5 ± 1.0 ^a^	9.7 ± 2.2 ^b^	1.7 ± 0.3 ^b^	15.5 ± 3.2 ^b^
Sc + Sk (99:1)	1.2 ± 0.5 ^a^	1.9 ± 0.9 ^a^	6.7 ± 1.2 ^a^	1.2 ± 0.3 ^a^	10.9 ± 2.2 ^a^
Sc + Sk_1_ (99:1)	1.6 ± 0.5 ^a,b^	2.2 ± 0.9 ^a^	7.8 ± 1.6 ^a,b^	1.6 ± 0.4 ^a,b^	13.0 ± 2.8 ^a,b^
Sc + Sk_3_ (99:1)	1.3 ± 0.4 ^a,b^	2.2 ± 0.6 ^a^	7.6 ± 1.6 ^a,b^	1.2 ± 0.2 ^a^	12.2 ± 2.3 ^a,b^
Sc + Sk_6_ (99:1)	1.8 ± 0.3 ^b^	2.1 ± 1.0 ^a^	9.1 ± 1.5 ^b^	1.7 ± 0.4 ^b^	14.7 ± 2.0 ^b^
Sig. ^1^	*	ns	**	*	**

The mean values with different letters (a,b) in the same column are significantly different (*p* < 0.05); “±” indicates standard deviation. ^1^ Sig.: significance; * and **—display the significance at 5 and 1% by least significant difference; ns: not significant.

**Table 5:** Mixed fermentation variants.

Code	Strain, Inoculation (Simultaneous or Sequential), Inoculum Ratio of Sc:Sk
Sc	*Saccharomyces cerevisiae* monoculture
Sk	*Saccharomyces kudriavzevii* monoculture
Sc + Sk (3:2)	simultaneous inoculation of Sc and Sk, inoculum ratio 3:2
Sc + Sk_1_ (3:2)	sequential inoculation of Sc followed Sk after one day, inoculum ratio 3:2
Sc + Sk_3_ (3:2)	sequential inoculation of Sc followed Sk after three days, inoculum ratio 3:2
Sc + Sk_6_ (3:2)	sequential inoculation of Sc followed Sk after six days, inoculum ratio 3:2
Sc + Sk (99:1)	simultaneous inoculation of Sc and Sk, inoculum ratio 99:1
Sc + Sk_1_ (99:1)	sequential inoculation of Sc followed Sk after one days, inoculum ratio 99:1
Sc + Sk_3_ (99:1)	sequential inoculation of Sc followed Sk after three days, inoculum ratio 99:1
Sc + Sk_6_ (99:1)	sequential inoculation of Sc followed Sk after six days, inoculum ratio 99:1

## Data Availability

Not applicable.
